# Effects of ginsenosides Re and Rg3 on intracellular redox state and cell proliferation in C6 glioma cells

**DOI:** 10.1186/1749-8546-3-8

**Published:** 2008-07-11

**Authors:** Wai Yee Ng, Mildred S Yang

**Affiliations:** 1 Department of Biology, Hong Kong Baptist University, Hong Kong SAR, PR China

## Abstract

**Background:**

Cellular redox state is important to cell growth and death. The growth of tumor cells may be modulated by intracellular reduced glutathione/oxidized glutathione (GSH/GSSG). The present study aims to investigate the effects of ginsenosides Re and Rg3 on cellular redox state and cell proliferation in C6 glioma cells.

**Methods:**

Cultured C6 glioma cells were exposed to various concentrations of either Rg3 or Re for 24 hours. Cell growth and death were measured by the BrdU incorporation assay and the 3-(4,5-dimethylthiazol-2-yl)-2,5-diphenyltetrazolium bromide (MTT) assay respectively. Cellular redox state was determined by free radical production using flow cytometry and GSH/GSSG using spectrofluorometry.

**Results:**

At a sub-lethal concentration, Re suppressed cell proliferation with a significant decrease in BrdU incorporation. Re did not increase reactive oxygen species (ROS) production but increased GSH/GSSG via increased activity of gamma glutamylcystenyl synthase (γ-GCS). In contrast, Rg3 increased free radical production and reduced GSH/GSSG. The effects of Rg3 were probably due to increased activity of glutathione peroxidase (GPx).

**Conclusion:**

Re and Rg3 alter cellular redox state of C6 glioma cells in opposite directions. Changes in cellular redox state induced by Re and Rg3 are correlated with the proliferation rates of C6 glioma cells.

## Background

Cellular redox state can be monitored by intracellular thiol levels, among which the ratio of reduced glutathione (GSH) and oxidized glutathione (GSSG) is the most useful [1,2]. In the presence of free radicals, mainly H_2_O_2_, GSH acts as electron donor and is oxidized into GSSG by glutathione peroxidase (GPx). The GSSG generated is later converted back to GSH by glutathione reductase (GR) in which reduced nicotinamide adenine dinucleotide phosphate (NADPH) is the hydrogen donor. In living tissues, the level of glutathione is ranged 5–10 mM. In cultured cells, the level of intracellular glutathione is several folds higher than the level of total adenosine nucleotides [3]. Under stable conditions, cells maintain a constant ratio of GSH/GSSG [1,4,5]. Excessive reactive oxygen species (ROS) decreases the GSH/GSSG ratio [6]. The GSH/GSSG ratio is increased by the addition of N-acetylcysteine (NAC, a cysteine analogue) [7] or by improved enzymes for glutathione synthesis [8]. Antioxidants or molecules that increase the GSH/GSSG ratio suppress cell proliferation [9-14], while oxidative stress increases cell proliferation and leads to cell death [2,15-19]. Cellular redox state also affects cellular signaling pathways, gene expression and enzymes associated with cell cycle progression [2,6,11,12,15,16,18,19]. Conour *et al. *identified 92 candidate proteins involved in cell cycle progression that are redox sensitive [20]. Growth and metastasis of tumor cells may also be regulated by intracellular redox state [2,17,21,22].

Ginseng (*Panax ginseng*, *Renshen*) is a popular Chinese herbal medicine known for its wide spectrum of pharmacological actions [23]. Ginseng is composed of a series of saponins known as ginsenosides which are derivatives of triterpene dammarane. There are two categories of ginsenosides, namely protopanaxadiol (PPD, e.g. Ra, Rb, Rc, Rd, Rg3, Rh2) and protopanaxatriol (PPT, e.g. Re, Rf, Rg1, Rg2, Rh1). Each gensenoside is characteristic in its sugar moiety position on dammarane. Some ginsenosides modulate cardiovascular and neurological functions [24], whereas others act as antioxidants [25-29] to prevent cancer [30-32], protect against chemically induced tissues damage [33-36] and delay ageing [25]. Ginsenoside Re has anti-oxidative abilities apart from its immunomodulatory, antihyperlipidemic and neuroprotective activities. In cardiomyocytes, pretreatment with Re significantly attenuates H_2_O_2 _induced free radical production and protect cell death [37]. Rg3 acts as a prooxidant to accelerate 2,2'-azobis (2-amidinopropane) hydrochloride (AAPH) induced haemolysis in human erythrocytes [26]. Rg3 is anti-angiogenic and anticancer through inducing apoptosis [30,38].

Ginsenosides are active components in ginseng which has anticancer properties [23,32,39-41]. The present study investigates how Re and Rg3 suppress the growth of C6 glioma cells. The C6 glioma cell is a model for studying a form of malignant brain tumor glioblastoma multiform [42]. The study also investigates the mechanisms through which ginsenosides Re and Rg3 modulate the levels of oxidized and reduced glutathione, and ROS production

## Methods

### Materials

Cell culture media and all reagents for biochemical assays were purchased from Sigma Chemical (MO, USA). Perchloric acid (PCA) and potassium hydroxide were purchased from Fisher Scientific (NJ, USA). Antibiotics were purchased from Life Technologies (NY, USA). Fetal bovine serum (FBS) and 6-carboxy-2,7-dichlorodihydrofluorescein diacetate (H_2_DCFDA) were purchased from Invitrogen (Scotland, UK) and Molecular Probes (NJ, USA) respectively.

**Figure 1:**
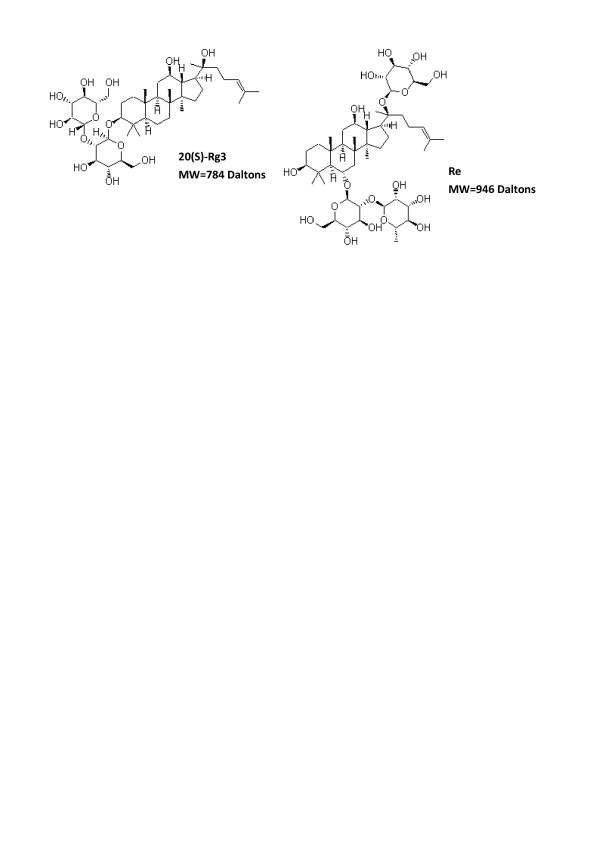
Chemical structures of ginsenosides Re and Rg3 (adapted from chemBlink ).

Ginsenosides Re and Rg3 (purity>99%) were obtained from International laboratory (USA). The identities of ginsenosdies were confirmed by high-performance liquid chromatography (HPLC) and electrospray ionization – mass spectrometry (ESI-MS). The Rg3, a 20(S) isoform, and the Re were 784 Daltons and 946 Daltons of molecular weight respectively (Figure 1). In this study, the concentration of ginsenosides was based on nominal weight. The ginsenosides were dissolved in dimethyl sulfoxide (DMSO), filtered (0.2 μm membrane) and added to the culture media to achieve various final concentrations.

Buthionine-(*S*, *R*)-sulfoximine (BSO), an inhibitor of gamma glutamylcystenyl synthase (γ-GCS), was dissolved in phosphate buffered saline (PBS) and sterilized through a 0.2 μm membrane. Cultured C6 glioma cells (ATCC no CCL-107) were purchased from the American Type Culture Collection (USA).

### Cell culture

The cells were maintained in the F-12 medium supplemented with 10% FBS (tested for lipopolysaccharide) and 0.5% antibiotics (amphotericin B and penicillin-streptomycin) in a humidifier under 95% air and 5% CO_2 _at 37°C.

### Treatment of cells

Cells were sub-cultured into 6-well plates until confluence. The incubation media were then removed and replaced with those containing the required concentration of Rg3 or Re. The concentration of DMSO in each well was kept at 0.5%, which did not affect intracellular glutathione level and GSH/GSSG. Cells were treated with 100 μM BSO for 24 hours for the study of cell response in the absence of GSH.

### Analysis of cell viability

The viability of C6 glioma cells treated with Re and Rg3 was determined by the 3-(4,5-dimethylthiazol-2-yl)-2,5-diphenyltetrazolium bromide (MTT) assay. Briefly, cells were cultured in 96-well plates and treated with various concentrations (0–800 μg/ml) of Re or Rg3. After incubation, cells were washed twice with PBS. A medium (100 μl) containing 500 μg/ml MTT was added to each well. The medium was removed after 3 hours of incubation and DMSO (100 μl) was added to dissolve the formazan crystals. Light absorbance at 595 nm was measured with a microplate reader (Tecan Infinite F200, Germany). Cell viability was the optical density ratio of a treated culture over an untreated control.

### Cell proliferation assay

The effects of either Re or Rg3 on cell proliferation were determined by the BrdU cell proliferation kit (Calbiochem, Germany). Cells were cultured in 96-well plates. Upon confluence, they were incubated with either Re or Rg3 of various concentrations for 24 hours. BrdU was added to the medium 20 hours before the end of incubation. The amount of BrdU incorporated into cells was determined by binding BrdU to mouse anti-BrdU antibody conjugated with horseradish peroxidase. Tetra-methylbenzidine, the substrate of horseradish peroxidase, was later added. The color product of the enzymatic reaction was measured in a microplate reader (Tecan Infinite F200, Germany) at 450–540 nm.

### Analysis of intracellular glutathione metabolism

Intracellular glutathione metabolism is reflected by the interplay of GSH, GSSG, GPx, GR and γ-GCS. Cells were incubated for 24 hours before intracellular metabolites were extracted by 0.3 M PCA containing 1 mM ethylenediamine-tetraacetic acid (EDTA) with a previously described method [43]. The levels of GSH and GSSG were determined with spectrofluorometry [3]. For enzyme assays, cells were washed twice with pre-warmed PBS and extracted with a protein extraction kit (EMB Biosciences, Calbiochem, USA) with a previously described method [8].

### Analysis of ROS production by flow cytometry

Intracellular production of ROS was evaluated with H_2_DCFDA which is converted to a fluorescence product dichlorodihydrofluoroscine (DCF) when treated with H_2_O_2_. Cells were treated for 24 hours with either 100 μg/ml of BSO or 200 μg/ml of Re. Thirty minutes before the end of experiment, H_2_DCFDA was added (final concentration 10 μM). The cells were then trypsinized, centrifuged, re-suspended in PBS and passed through the FACScan flow cytometer (Becton Dickinson, U.S.A.). Fluorescence intensities of DCF were measured by excitation wavelength of 488 nm and emission wavelength of 620 nm. In each sample, approximately 10000 cells were analyzed by the CellQuest software (Becton Dickinson, U.S.A.).

### Data analysis

Cellular redox state was determined by the ratio of GSH/GSSG. Each experiment was repeated 3–8 times. The means and standard deviations were calculated. The differences between samples were evaluated by the one way analysis of variance (ANOVA), followed by tests for differences between groups by a post-hoc method (Dunnett C) without an assumption of equal variance between groups (SPSS version 14). The differences with P < 0.05 were considered statistically significant.

## Results

### Selection of concentrations of Re and Rg3 for the study of cell proliferation

**Figure 2:**
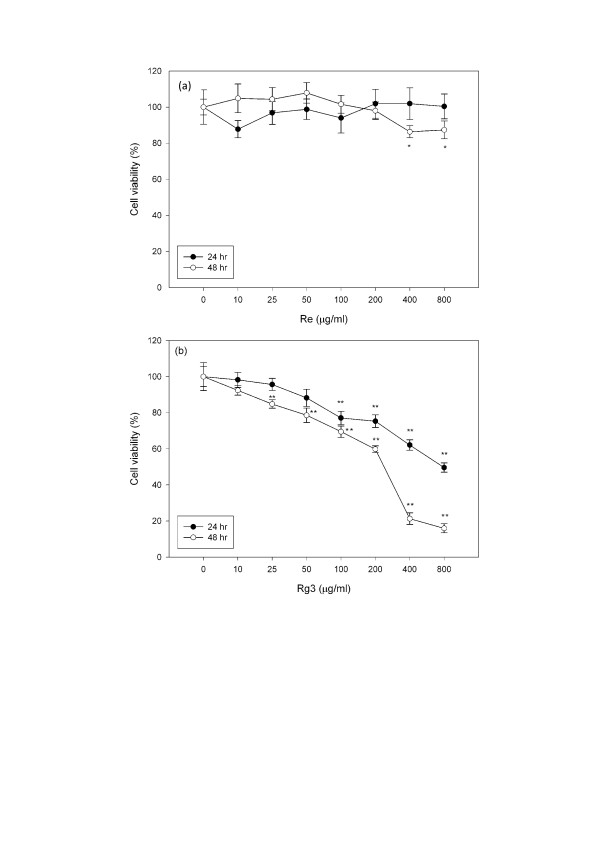
**Changes in cell viability of cells treated with (a) Re or (b) Rg3 for 24 and 48 hours**. Each value represents the mean and standard deviation of 8 replicates. **: statistically significant (P < 0.01)

A 24 and 48 hour dose-response study was conducted. Cells were treated with various concentrations (0–800 μg/ml) of Re and Rg3. There was a significant reduction (P < 0.01) in cell viability upon treatment with 100 μg/ml of Rg3 for 24 hours (Figure 2). At 48 hours, a significant difference (P < 0.01) in cell viability was observed when Rg3 concentration was 50 μg/ml. In contrast, there was no significant change in cell viability after treatment with Re for 24 hours. At high concentrations (400 and 800 μg/ml), cell viability decreased 20% after 48 hours.

**Figure 3:**
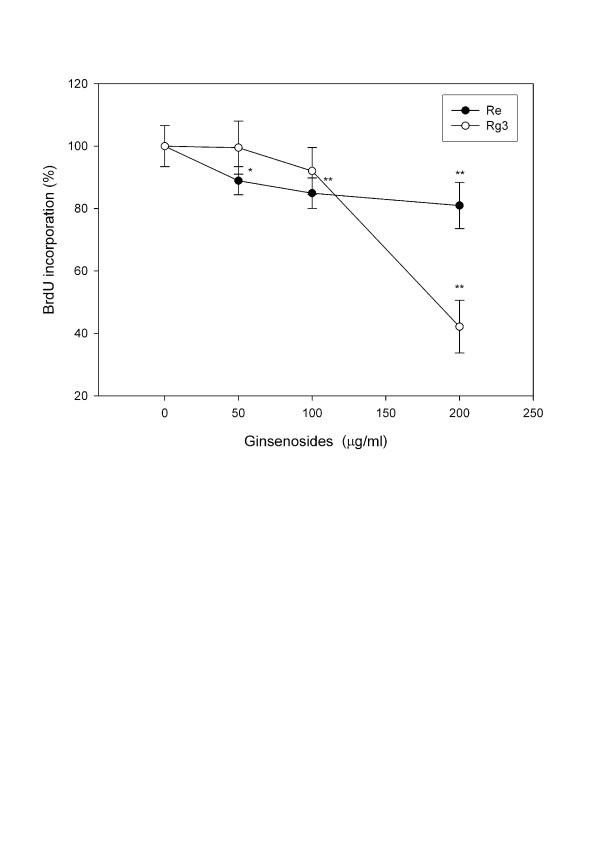
**BrdU incorporation assay in C6 glioma cells treated with various concentrations of Re and Rg3 for 24 hours**. Each value represents the mean and standard deviation of 8 replicates. *: statistically significant (P < 0.05). **: statistically significant (P < 0.01).

A decrease in cell proliferation was also demonstrated by decreased BrdU incorporation. While cell viability did not decrease (Figure 2a) after treatment with low concentrations of Re (50–200 μg/ml), BrdU incorporation (Figure 3) decreased 20% (P < 0.01). In contrast, Rg3 did not decrease BrdU incorporation at concentrations below 200 μg/ml at which cell viability significantly decreased (P < 0.05).

### Glutathione metabolism

**Figure 4:**
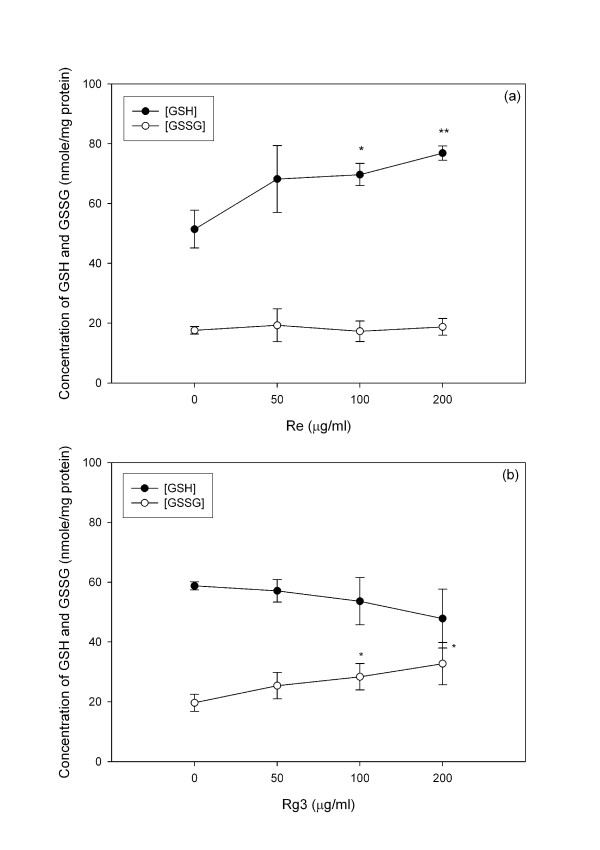
**Changes in the GSH and GSSG levels upon treatment with (a) Re or (b) Rg3 for 24 hours**. Each value represents the mean and standard deviation of 4 replicates. *: statistically significant (P < 0.05). **: statistically significant (P < 0.01).

**Figure 5:**
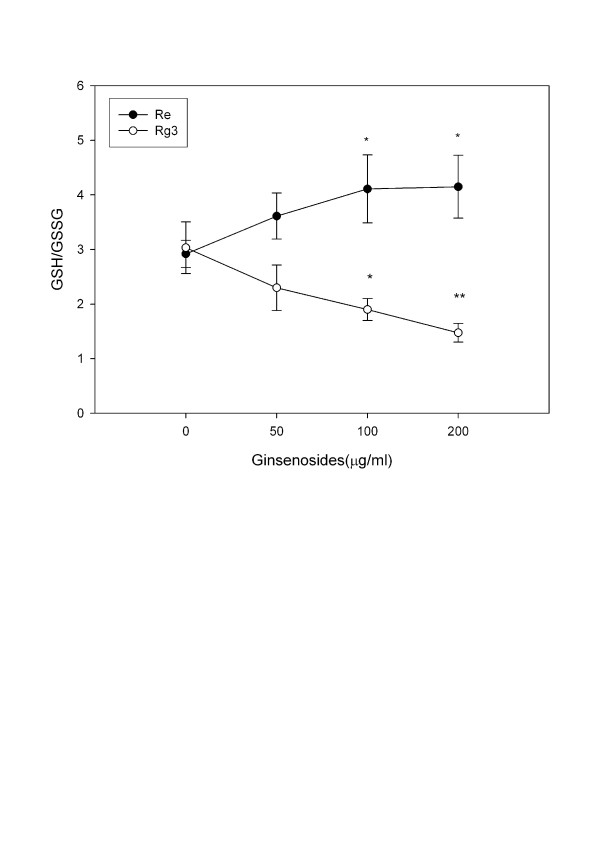
**Change in the GSH/GSSH ratio of C6 glioma cells treated with Re and Rg3 for 24 hours**. Each value represents the mean and standard deviation of 4 replicates. *: statistically significant (P < 0.05). **: statistically significant (P < 0.01).

**Figure 6:**
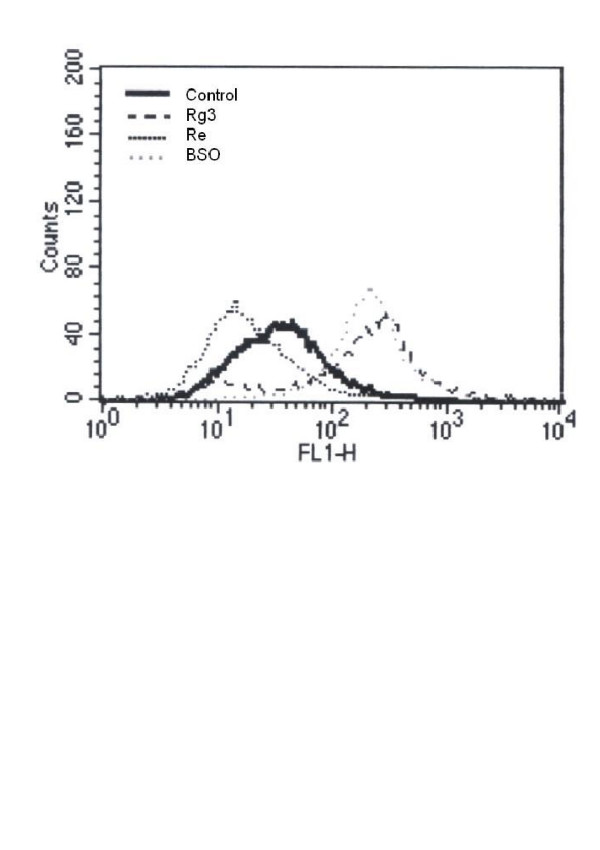
**Changes in the intracellular ROS level indicated by the fluorescence intensity (FLH-1) in C6 glioma cells treated with Re (200 μg/ml), Rg3 (200 μg/ml) or BSO (100 μM) for 24 hours**. The cytogram represents three independent experiments. The x-axis and y-axis denote fluorescence intensity and cell count respectively.

The resting level of GSH is three folds higher than that of GSSG. The GSH/GSSG ratio ranges from 2 (in tumor cells) to >40 (in normal cells) [44]. The GSH/GSSG ratio may be altered by either toxicants [3] or antioxidants [45]. Re treatment increased GSH but not GSSG (Figure 4a), while Rg3 increased GSSG (Figure 4b) but not GSH. Rg3 treatment decreased the GSH/GSSG ratio, while Re treatment increased it (Figure 5). A decrease in the GSH/GSSG ratio after treatment with Rg3 indicated oxidative stress. ROS production was increased after treatment with Rg3 (Figure 6). Rg3, which decreased the GSH/GSSG ratio at 200 μg/ml, also significantly increased ROS generation (Figure 6). The results show that Rg3 and Re treatments changed the intracellular GSH/GSSG ratio in opposite directions.

**Figure 7:**
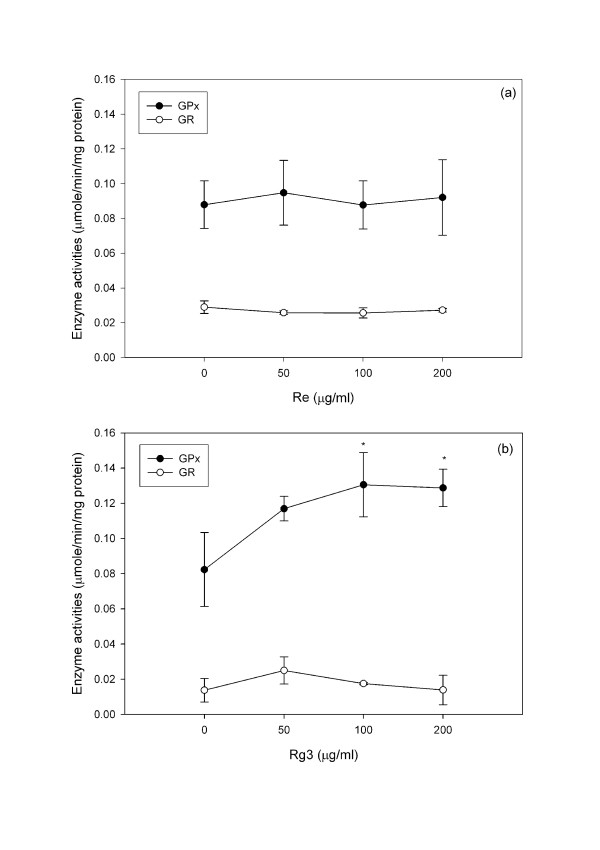
**Changes in the GR and GPx activities of C6 glioma cells treated with (a) Re and (b) Rg3 for 24 hours**. Each value represents the mean and standard deviation of 4 replicates. *: statistically significant (P < 0.05).

**Figure 8:**
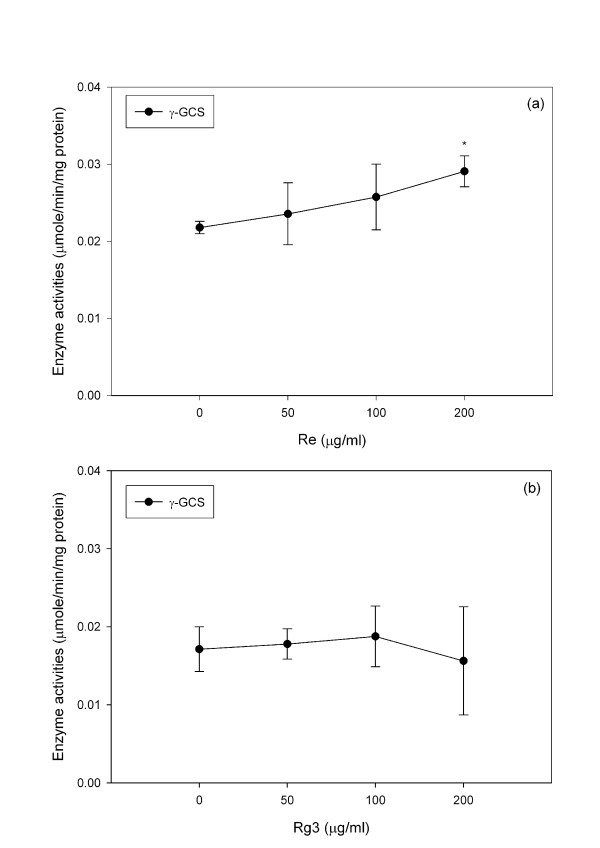
**Changes in the γ-GCS activity of C6 glioma cells treated with Re and Rg3 for 24 hours**. Each value represents the mean and standard deviation of 4 replicates. *: statistically significant (P < 0.05).

Re treatment did not significantly change the activity of either GPx or GR (Figure 7a) but increased (P < 0.05) the activity of γ-GCS, which is the rate limiting enzyme for GSH synthesis (Figure 8). Rg3 significantly increased (P < 0.05) GPx without changing GR (Figure 7b) or γ-GCS (Figure 8).

### Suppreession of γ-GCS activity by BSO reverses Re induced changes

**Figure 9:**
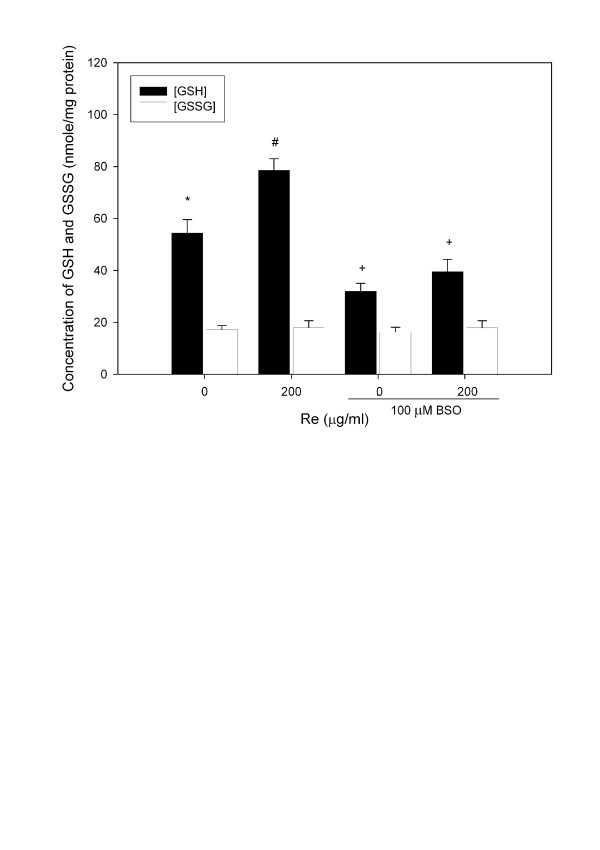
**Changes in the GSH and GSSG levels upon treatment with Re and/or BSO for 24 hours**. Each value represents the mean and standard deviation of 4 replicates. Symbols indicate that the treatment groups are significantly different (P < 0.05) from each other.

**Figure 10:**
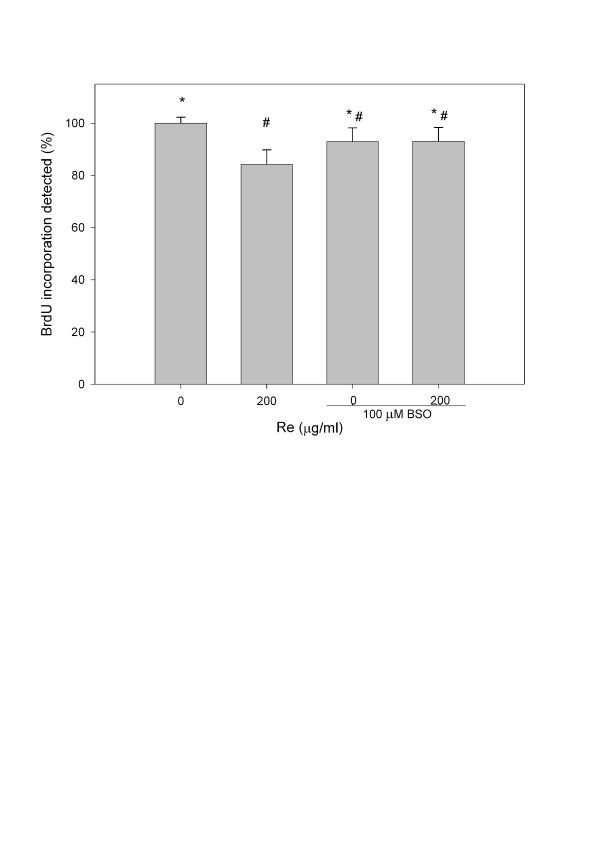
**BrdU incorporation assay in C6 glioma cells treated with Re and BSO for 24 hours**. Each value represents the mean and standard deviation of 4 replicates. Symbols indicate that the treatment groups are significantly different (P < 0.05) from each other.

To further confirm that Re acts by changing γ-GCS activity, treatment of cells with BSO, an inhibitor of γ-GCS which could significantly lowered (P<0.01) the GSH/GSSG ratio (Figure 9) and increased (P<0.05) cellular ROS production (Figure 6), was able to reverse the Re induced change in both GSH/GSSG (Figure 9) and BrdU incorporation (Figure 10).

## Discussion

The present study showed that both Re and Rg3 affected the survival of C6 glioma cells. While Re suppressed the growth of C6 glioma cells at low concentrations, Rg3 caused death of C6 glioma cells via oxidative stress. Furthermore, we demonstrated that the action of Re was correlated with an increase in the intracellular GSH level, thereby raising the GSH/GSSG ratio. BSO (an inhibitor of γ-GCS) treatment reversed the GSH/GSSG rise induced by Re (Figure 9) and decreased BrdU incorporation in the presence of Re (Figure 10).

γ-GCS is the rate limiting enzyme in glutathione synthesis. The enzyme and its gene expression may be modulated by a number of factors. For example, an increase in the γ-GCS activity may be stimulated by oxidative metabolites. Shi *et al. *showed that the heavy subunit mRNA level and subsequent enzyme activity increased in response to 2,3-dimethoxy-1,4-napthoquinone (DMNQ) through transcriptional activation of γ-GCS [46]. Our previous studies showed that the production of quinone metabolite of benzo [a]pyrene activated γ-GCS [8]. However, no such quinone metabolites were detected in the metabolism of Re in biological tissues. Furthermore, the present study showed that Re treatment suppressed free radical production (Figure 6), which did not support the free radical production hypothesis in γ-GCS activation. Another study showed that PC12 cells' tolerance of 7-hydroxycholesterol and 15-deoxy-12,13- prostaglandin J2 was achieved via up-regulation of cellular glutathione by γ-GCS transcription [47]. Such feedback activation may be an underlying cause of cellular adaptation to oxidative stress. Therefore, similar adaptive response may take place in C6 glioma cells following Re treatment.

A number of factors must be considered before using Re to treat brain tumor. Firstly, Re suppresses cell proliferation at a low rate. At a low concentration of 50 μg/ml, Re was effective in suppressing cell proliferation (Figure 3). However, there was only a 10% decrease in BrdU uptake after 24 hours (Figure 2a), and only a 20% decrease in cell viability after 48 hours. Secondly, Re is metabolized by intestinal flora via deglycosylation and fatty acid esterification [48], which may limit the application of Re given *per oral*. Finally, it is not clear whether normal astrocytes respond similarly to Re. In current anticancer strategies, both normal and tumor cells are affected. Menon *et al. *demonstrated that exposure of both nonmalignant human breast epithelial (MCF-10A) and breast cancer cells (MCF-7 and MDA-MB-231) to NAC decreased the oxidation of a prooxidant-sensitive dye in MCF-10A cells, but not in MCF-7 and MDA-MB-231 cells, suggesting that malignant and non-malignant cells may have different responses to oxidative stress [17]. We will further investigate whether C6 glioma cells and normal astrocytes respond differently to Re treatment.

## Conclusion

Re suppresses the growth and proliferation of C6 glioma cells and increases the cellular GSH/GSSG ratio by enhancing the γ-GCS activity, suppressing ROS generation and reducing cell proliferation rate. Rg3 causes cell death via oxidative stress. While both Re and Rg3 eliminate the growth and proliferation of C6 glioma cells, they modulate cellular redox state in opposite directions. Yue *et al. *suggest that ginseng or selected ginsenosides modulate cellular function via specific intracellular receptors in a way that may reflect the Ying/Yang actions of ginseng [49]. The opposite actions in modulating cellular redox state by Re and Rg3 may be another example of the Ying/Yang actions of ginseng.

## Abbreviations

γ-GCS: gamma glutamylcystenyl synthase; AAPH: 2,2'-azobis (2-amidinopropane) hydrochloride; BSO: buthionine-(*S*, *R*)-sulfoximine; DMNQ: 2,3-dimethoxy-1,4-napthoquinone; DMSO: dimethyl sulfoxide; EDTA: ethylenediamine-tetraacetic acid; ESI-MS: electrospray ionization – mass spectrometry; Fetal bovine serum (FBS); GPx: glutathione peroxidase; GR: glutathione reductase; GSH: reduced glutathione; GSSG: oxidized glutathione; H_2_DCFDA: 6-carboxy-2,7-dichlorodihydrofluorescein diacetate; HPLC: high-performance liquid chromatography; MTT: 3-(4,5-dimethylthiazol-2-yl)-2,5-diphenyltetrazolium bromide; NAC: N-acetylcysteine; NADPH: nicotinamide adenine dinucleotide phosphate; -PBS: phosphate buffered saline; Perchloric acid (PCA); PPD: protopanaxadiol; PPT: protopanaxatriol; -ROS: reactive oxygen species

## Competing interests

The authors declare that they have no competing interests.

## Authors' contributions

WN is an MPhil student who carried out the experiments and helped draft the manuscript. MY supervised the study and drafted the manuscript. Both authors read and approved the final version of the manuscript.
